# Experimental Evaluation of an Array Transducer for Ultrasound Thermal Strain Imaging: Phantom and *In Vivo* Studies

**DOI:** 10.1016/j.ultrasmedbio.2025.05.016

**Published:** 2025-06-07

**Authors:** Zhiyu Sheng, Ran Wei, Mengyue Chen, Matthew B. Wielgat, Dhanansayan Shanmuganayagam, Edith Tzeng, Xuecang Geng, Xiaoning Jiang, Kang Kim

**Affiliations:** a Department of Medicine, University of Pittsburgh, Pittsburgh, PA, USA; b Department of Bioengineering, University of Pittsburgh, Pittsburgh, PA, USA; c Department of Mechanical and Aerospace Engineering, North Carolina State University, Raleigh, NC, USA; d Department of Animal and Dairy Sciences, University of Wisconsin-Madison, Madison, WI 53706, USA; e Department of Surgery, UW School of Medicine and Public Health. University of Wisconsin-Madison, Madison, WI, USA; f Department of Surgery, University of Pittsburgh, Pittsburgh, Pittsburgh, PA, USA; g Blatek Industries, Inc., Boalsburg, PA, USA; h Department of Mechanical Engineering and Materials Science, University of Pittsburgh, Pittsburgh, PA, USA; i Heart and Vascular Institute, University of Pittsburgh Medical Center (UPMC), Pittsburgh, PA, USA; j Vascular Medicine Institute, University of Pittsburgh, PA, USA; k McGowan Institute of Regenerative Medicine, Pittsburgh, PA, USA; l Department of Biomedical Engineering, Carnegie Mellon University, Pittsburgh, PA, USA

**Keywords:** Atherosclerosis plaque (AP), Medical ultrasound imaging, Ultrasound thermal strain imaging (US-TSI), Temperature measurements

## Abstract

**Objective:**

Characterization of atherosclerosis plaque (AP) is critical for diagnosing rupture-prone AP that directly causes stroke and heart attack, and for guiding in-time interventions and avoiding unnecessary surgeries for stable cases. Ultrasound thermal strain imaging (US-TSI) is known to be capable of characterizing lipids, an important feature of rupture-prone AP. However, before translating US-TSI to *in vivo* clinical applications, significant technical challenges must be overcome, primarily the requirements of a well-controlled heating strategy to achieve a rapid, safe and spatial-temporal−precise local tissue temperature increase.

**Methods:**

To address these issues, we recently developed a novel US-TSI transducer that integrates dual ultrasound heating arrays that use the thermal effect of the acoustic radiation force, and an ultrasound imaging array to reconstruct the spatial thermal strain map.

**Results:**

This article presents the first comprehensive test results of our new US-TSI transducer including benchtop US-TSI experiments on ultrasound gelatin phantoms with spatial temperature measurements to compare the thermal strain pattern and the corresponding 2-D temperature map, and US-TSI experiments on a pig with temperature measurements to verify the *in vivo* feasibility and safety further. A clear thermal strain pattern was obtained as a maximum of −0.25% in phantom and −0.08% *in vivo*, which corresponds with a reasonable temperature increase, 2.5°C in the phantom and 0.9°C *in vivo*. There was also a high resemblance between the thermal strain pattern and corresponding temperature measurements.

**Conclusion:**

The results demonstrate the effectiveness and safety of performing US-TSI using our new array transducer.

## Introduction

Atherosclerosis is related to many diseases including myocardial infarction, stroke, abdominal aneurysms and lower limb ischemia that are associated with high morbidity and mortality [1]. Among these, rupture of atherosclerosis plaque (AP) that directly causes a heart attack and stroke is the most acute adverse [2]. It is therefore critical to understand and diagnose the high-risk rupture-prone, or unstable, vulnerable AP from stable AP to provide timely interventions while avoiding unnecessary surgeries for the stable cases. Studies showed that high-risk AP could be characterized by a thin or almost absent fibrous cap, high lipid concentration, plaque inflammation-related activities like macrophage and T-cell recruitment, intraplaque hemorrhage, vasculature remodeling and neovascularization, and so on [3]. Although intracoronary imaging techniques, such as intravascular ultrasound [4], near-infrared spectroscopy [5,6], optical coherence tomography, and micro-optical coherence tomography [4,7–9] have contributed to assess these features, ultrasound imaging, considered completely noninvasive, nonionizing and relatively low cost, is often preferred during regular clinical screenings. However, the current clinical standard for ultrasound examinations is combined B-mode and Doppler imaging, which only visually approximates the stenosis based on general morphology and blood flow information, and is not specific for characterizing the composition of the AP. Therefore, it cannot accurately identify rupture-prone AP and suggest the optimal treatment: vulnerable AP is not necessarily associated with significant stenosis.

To predict rupture-prone AP, there have been emerging quantitative ultrasound imaging technologies aiming at tissue characterization based on different ultrasound physics. For example, the gray-scale median score assesses plaque vulnerability based on echogenicity [10]. To investigate tissue properties more directly, ultrasound vascular elastography, including both transcutaneous [11] and intravascular approaches [12], uses pulsation as the excitation and analyzes the displacements and strains to characterize AP via a relative mechanical contrast. Acoustic radiation force impulse (ARFI) imaging uses ARF as a well-controlled and location-specific excitation for tissue characterization, as indicated by the push-induced mechanical displacement contrast [13,14]. Compared with these techniques, ultrasound thermal strain imaging (US-TSI) [15–22] has been proposed to focus specifically on lipid identification based on the contrast owing to the opposite speed of sound (SoS) change in lipids compared with water-bearing tissues when temperature increases. It is well-known that the SoS increases in water and decreases in fat when temperature increases. According to this principle, US-TSI derives the thermal strain by first heating the target area, then imaging and estimating the spatial echo shifts, and finally computing the axial gradient. As a result, local SoS decrease or increase can be inferred from a negative or positive gradient, to contrast the lipid contents of water-bearing tissue, respectively.

Lipid identification using US-TSI has previously been reported in studies of fatty livers and AP [17,23]. However, for translating US-TSI to *in vivo* clinical applications, especially for the cardiovasculature, there are still unaddressed technical challenges including the requirement of a well-controlled heating strategy to precisely achieve a safe temperature increase (<2°C), and the difficulty of separating the extremely small thermal-induced echo shifts in the presence of larger mechanical motions owing to cardiac pulsation and respiration. To facilitate this difficulty, potential solutions either require performing US-TSI in a very short time interval so that mechanical motions of finite speed do not integrate to a large amplitude or need an electrocardiogram (ECG) or image-based gating techniques [24,25] so that the frames to compute thermal strain are approximately relatively static. Both solutions require a rapid heating process. The requirement is obvious for the former while rapid heating is also highly desired for the latter because it enables the gated image frames to be within only one or two cardiac cycles to mitigate decorrelation owing to other unknown motion sources.

To address these technical hurdles, we recently developed a novel transcutaneous US-TSI transducer that integrates dual ultrasound heating arrays and an ultrasound imaging array for carotid atherosclerosis. Owing to the thermal effect of the ARF [26], the heating arrays can rapidly and precisely deliver controlled acoustic energy to the focal area that is aligned with the region of interest of the imaging array. The heating arrays use PZT-5A as the active layer owing to its superior electrical impedance and transmitting sensitivity. They are designed to have the capability of increasing tissue temperature by 2°C within 50 ms, which has been presented in our previous works that were focused on the design and test of the heating arrays [27–29]. Comprehensive tests to assess performance and safety must be performed before using the developed prototype for *in vivo* preclinical human studies. This paper presents the first test results of this US-TSI transducer including benchtop experiments on ultrasound gelatin phantoms with spatial temperature measurements to compare the thermal strain pattern and the corresponding 2-D temperature map, and US-TSI experiments on an anesthetized pig with temperature measurements to verify the *in vivo* feasibility and safety further.

## Materials and methods

### Transducer prototype and experiment sequence

**Figure 1.**
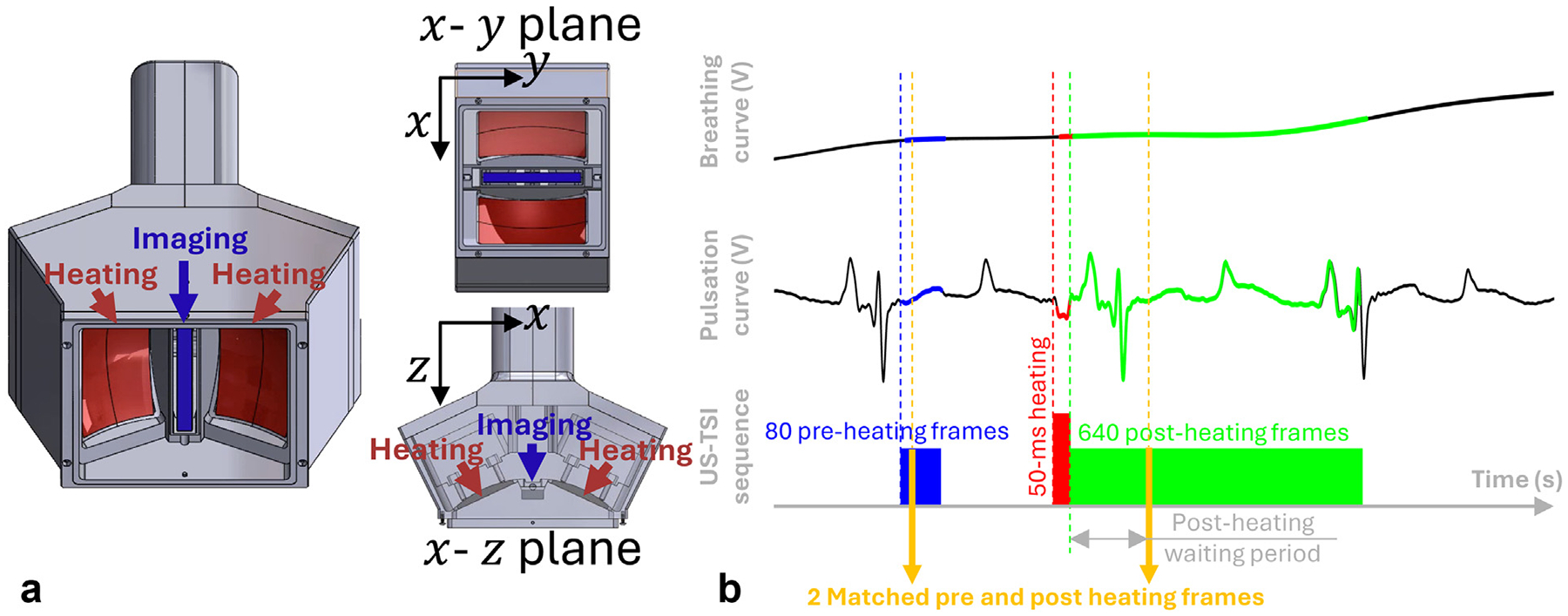
(a) The Ultrasound thermal strain imaging (US-TSI) array transducer prototype that contains dual heating arrays that use the thermal effect of acoustic radiation force impulse and the imaging array that provides a field of view in the y-z plane. (b) US-TSI experiment sequence. Preheating frames are marked in *blue* and the post-heating frames are marked in *green*. The heating duration is marked in red. During *in vivo* US-TSI experiments, electrocardiogram (ECG)-based triggering and gating were applied based on the breathing and pulsation curves. During phantom experiments, because the ECG-based triggering and gating is not applicable, the preheating, heating, and post-heating intervals were consecutive without time gaps.

Figure 1a illustrates the prototype of the US-TSI transducer that integrates an imaging array and dual heating arrays. The imaging array has a bandwidth of 6−13 MHz. It contains 192 elements with a 0.20 mm pitch and provides a field of view (FOV) in the y-z plane. The heating arrays have a center frequency of 3.5 MHz considering the frequency-dependent absorption coefficient that determines both penetration and thermal effect. Along the x direction, the heating arrays contain 16 × 2 elements (16 on each side) with a 1.03 mm pitch. Each element is concave in both x and y directions, so that if all the elements transmit simultaneously, a geometric focus will be formed at z=25mm in the image FOV (y-z plane). To increase the size of the focal region, we applied additional element phase delay electrically, although the peak intensity at focus is expected to decrease slightly. The integrated US-TSI transducer is connected to a Vantage 256 HIFU (Verasonics Inc., Kirkland, WA, USA) ultrasound system through a UTA-408 adapter (Verasonics Inc.) with an external QPX600DP power supply (Aim and Thurlby Thandar Instruments, Ltd., Huntingdon, UK).

During US-TSI experiments (Fig. 1b), we programmed to first acquire 80 preheating image frames using five-angle compounded planewave (evenly spaced from −10° to 10°) with a frame rate of 500 Hz, and then execute the heating sequence, immediately followed by 640 post-heating frames using the same acquisition parameters. By theory, thermal strain imaging needs only one preheating frame and one post-heating frame to detect the echo shifts between them. However, in practice, we decided to collect a series of frames (80 preheating frames and 640 post-heating frames). For *in vivo* pig experiments, this enabled frame selection by applying ECG gating to mitigate mechanical motion artifacts owing to cardiac pulsation and respiratory motions. For phantom experiments, the series of frames made it possible to apply a finite impulse response low-pass filter (transition band from 4 to 10 Hz) to remove standing and reflecting mechanical waves (owing to limited phantom dimensions and container boundaries). The heating sequence lasted for 50 ms with a 240 cycle long pulse repeating with a 50% duty cycle.

### Simulations of the expected temperature increase

**Figure 2.**
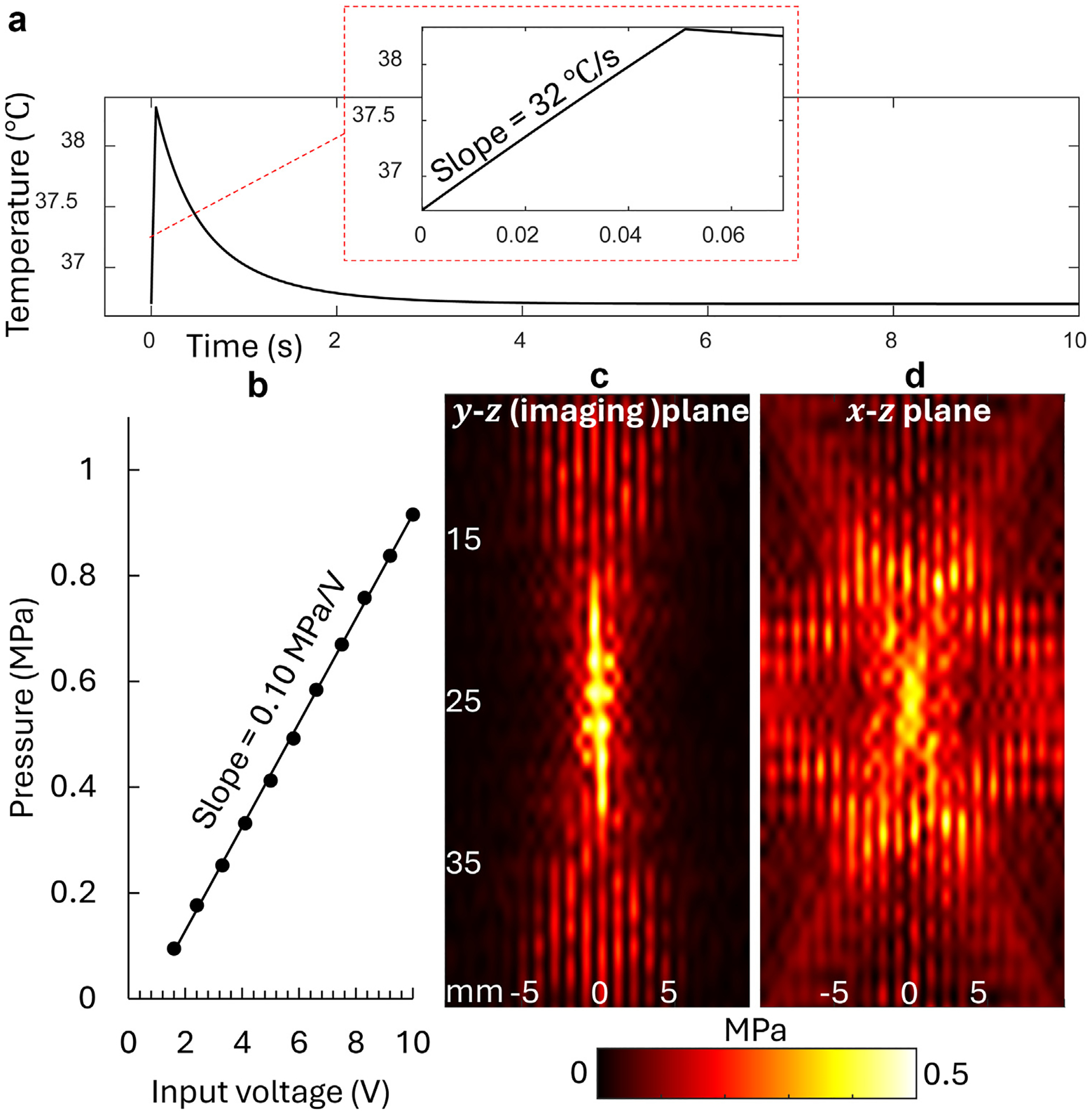
(a) Simulated temperature response at focus when dual heating arrays transmit with duty cycle of 50% and $P_{\text{RMS}}^{2}=8(\text{MPa})$ [2]. (b) Heating array transmit sensitivity at focus. (c and d) Heating array transmit pressure field in the y-z plane (b) and x-z plane (c).

To better understand and design the heating energy delivery by the heating arrays, we performed simulations of the expected temperature increase pattern owing to the ARF thermal effect. The simulation was performed on a rectangular homogeneous media based on the diffusion-convection equation of the spatial−temporal temperature distribution, u(x,y,z,t):

$$\frac{\partial u(x,y,z,t)}{\partial t}=\frac{\partial }{\partial x}\left(D_{x}(x,y,z)\frac{\partial u(x,y,z,t)}{\partial x}\right)+\frac{\partial }{\partial y}\left(D_{y}(x,y,z)\frac{\partial u(x,y,z,t)}{\partial y}\right)\;+\frac{\partial }{\partial z}\left(D_{z}(x,y,z)\frac{\partial u(x,y,z,t)}{\partial z}\right)-v_{x}\left(x,y,z\right)\frac{\partial u\left(x,y,z,t\right)}{\partial x}+\frac{Q\left(x,y,z,t\right)}{\rho \left(x,y,z\right)c_{h}\left(x,y,z\right)},$$

where velocity $v_{x}=0$ when there is no flow, ρ is the density, $c_{h}$ is the specific heat capacity, and $D_{x},D_{y}$, and $D_{z}$ are thermal diffusivity constants, computed as $\frac{k_{t}}{\rho c_{h}}$. Q is the input heat rate per unit volume and is the result of ARF thermal effect, as $Q=\gamma \frac{2\alpha P_{\text{RMS}}^{2}}{\rho c}$, where, γ is the duty cycle, α is the absorption coefficient, c is the SoS, and $P_{\text{RMS}}$ is the root mean square (RMS) value of the acoustic pressure. We approximated the spatial temperature gradient by finite difference and transformed the diffusion-convection equation to a linear time invariant system in the form of $\frac{du(t)}{dt}=Au(t)+q(t)$, where $u=\left[u_{i,j,k}\right]$ is a column vector of discretized temperature at different spatial locations and $q=\left[q_{i,j,k}\right]$ is the source column vector determined by Q and boundary conditions. We applied Neumann boundary condition at the top surface (which represents skin surface) as ${\left.\frac{\partial u}{\partial z}\right|}_{z=0}=0$ to assume insulation and applied Dirichlet boundary conditions for other surfaces as u=36.7°C (body temperature). Given the simulation parameters [30,31], as $k_{t}=0.484\text{W}/(\text{m}\cdot \text{K})$, $\rho =1000\;\text{kg}/{\text{m}}^{3},\;c=1540\;\text{m}/\text{s},\;c_{h}=3750\;\text{J}/(\text{kg}\cdot \text{K})$, α=8Np⋅3.5MHz,γ=50% and the pressure field $P_{\text{RMS}}$ computed by COMSOL Multiphysics (6.1, COMSOL Inc., Stockholm, Sweden), the linear time invariant system is easy to solve and provides the simulated temperature at focus as shown in Figure 2a. We can observe the heating process during the first 50 ms, when heating arrays are transmitting, followed by the cooling process when heating arrays are off.

It is important to note that the temperature curve during the 50 ms heating process is approximately linear with a slope close to the value of $\gamma \frac{2\alpha P_{\text{RMS}}^{2}}{\rho^{2}cc_{h}}$. This indicates that, in the 50 ms interval, the diffusion effect is negligible, and the temperature increase pattern is directly related to the heat source pattern that is determined by the acoustic pressure field pattern. Therefore, we can tune the maximum (at focus) value of $P_{\text{RMS}}^{2}$ to be 8 (MPa)^2^ so that the temperature can achieve the 2°C increase in 50 ms.

### Hydrophone measurements

According to the simulation, the magnitude of $P_{\text{RMS}}$ directly determine the expected magnitude of the temperature increase, which determines the capability of performing US-TSI. In addition, we concluded that $P_{\text{RMS}}^{2}$ should be approximately 8 (MPa)^2^ to satisfy the power requirement given a 50% transmit duty cycle. Note that (given the frequency of 3.5 MHz) $P_{\text{RMS}}^{2}=8(\text{MPa}{)}^{2}$ results in a safe mechanical index of 1.5 < 1.9. Further, we conducted hydrophone measurements to obtain the actual transmit capability reflected by transmit sensitivity at focus and the beam pattern. A calibrated bullet hydrophone (HGL-0085, ONDA Corporation, Sunnyvale, CA, USA) was utilized to scan the acoustic pressure field of US-TSI array transducer with a three-axis motion stage. The step size was 0.5 mm along both two horizontal axes, and 1.0 mm along the vertical axis. Using the Verasonics ultrasound system, a sinusoidal pulse with 10 cycles every 1 ms was generated to drive the US-TSI array transducer, while a digital oscilloscope (DSO7104B, Agilent Technologies, Inc., Santa Clara, CA, USA) was chosen to record the signal from the hydrophone. The RMS of the signal at each location was then computed and co-registered to form an acoustic pressure field map. To calibrate the transmit sensitivity, we measured RMS pressure values at focus associated with different transmit voltages, 1.6, 2.4, 3.3, 4.1, 5, 5.8, 6.6, 7.5, 8.3, 9.2, and 10 V. The transmit sensitivity was then derived by the slope of the RMS pressure-voltage curve.

The measurement results are summarized in Figure 2a, 2b, 2c, and 2s. It is observed from Figure 2b that the transmitting sensitivity is 0.10 MPa/V. Based on this, it can be approximated that when transmitting at a higher voltage like 50 V, the maximum pressure is higher than needed to achieve the desired slope, $\gamma \frac{2\alpha P_{\text{RMS}}^{2}}{\rho^{2}cc_{h}}$, which is approximated by the previous simulation. Figure 2c and 2d show the measured acoustic (RMS) pressure field in the x-z and y-z planes, respectively, using an input voltage of 10 V. The colormap was saturated to the range of 0 to 0.5 MPa for a better visualization. Considering both y-z plane and x-z planes, and the full width half maximum, the pressure field pattern indicates an expected temperature increase region of 10mm(z) by 2mm(y) centered at z = 25 mm in the imaging (y-z) plane. Note that our measurements are in a low amplitude range. A more accurate estimation for higher transmit power that considers nonlinear effect at focus will require methods as described in these studies [32,33].

### Image processing of US-TSI

Inside the image FOV (z-y plane), the thermal strain image, ε(z,y), can be determined by local tissue characteristics, λ, and temperature change, ΔT, as ε=-λΔT. For a well-controlled ΔT, the fact that λ<0 in fat and λ>0 in water makes it feasible to contrast lipid contents with water-bearing tissue. The derivations are adapted from the work [34] and are reviewed in detail in the supplementary materials.

To obtain ε(z,y), we first performed delay-and-sum beamforming and then computed the thermal displacement between the reference preheating frame and pos-heating frames by the Loupas’ tracking algorithm [35]. Note that, because we only considered the echo shifts in the z-axis, to approximately resemble the case in practice, the f-number during beamforming is set to at least 1.4, so that the angles of the pathways to the z-axis during transmission are at most 20°. Finally, the thermal strain image is the spatial gradient of the thermal displacement between the reference and the selected post-heating frame, computed by applying a 2-D Savitzky-Golay filter [36] that performs smoothed differentiation in the axial direction and smoothing in the lateral direction. For *in vivo* case, the selection of the reference preheating frame and the matched post-heating frame was based on ECG-gating. For phantom experiments, the last preheating frame was used as the reference while the 100th post-heating frame was selected after the transient state of the applied finite impulse response filter.

### Ultrasound gelatin phantom experiments

**Figure 3.**
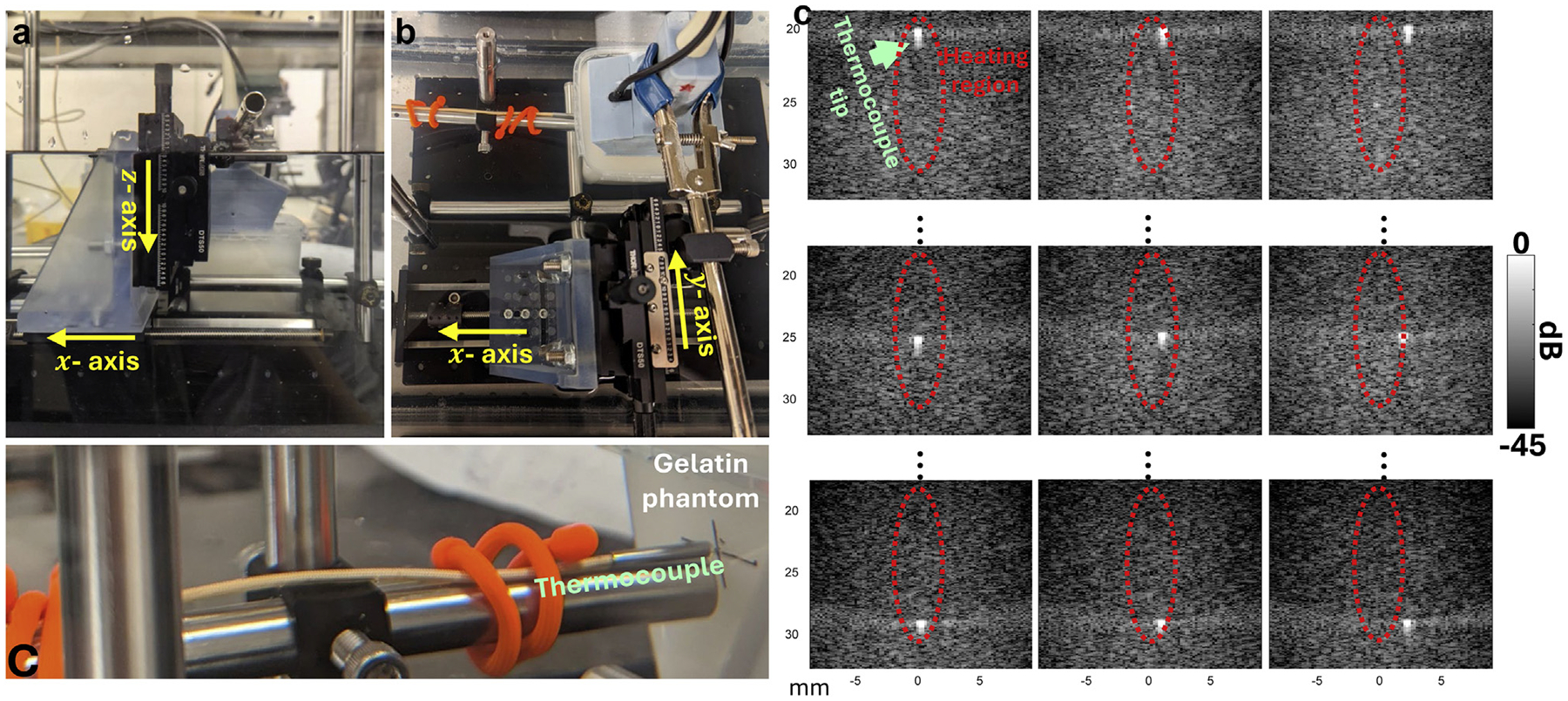
(a, b, and c) The underwater experiment setup for performing ultrasound thermal strain imaging (US-TSI) on gelatin phantoms with thermocouple measurements. The US-TSI array transducer can be adjusted by the three-axis translational stage so that the thermocouple can measure temperature increases at different relative locations to the heating focus. (d) Ultrasound B-mode images to demonstrate different relative positions of the thermocouple with respect to the heating region that is circled by the *red dashed sketch*.

There are two objectives for benchtop US-TSI experiments on ultrasound gelatin phantoms. The first objective is to compare the thermal strain pattern and the corresponding 2-D temperature map in image FOV. We performed the experiments underwater (Fig. 3), where the US-TSI transducer was mounted on a three-axis translational stage and a fast-response (25-ms time constant) thermocouple (MT29, Physitemp Instruments, LLC, Clifton, NJ, USA) was inserted (along the x-axis) into the gelatin phantom at approximately 10 mm beneath the surface. Specifically, we first located the x-axis of the translational stage at the position to best view the thermocouple tip and then translated the y-axis and z-axis to create mesh grid locations, (z,y), at z=19,21,23,25,27, and 29 mm and y=0,1, and 2 mm. At each location, we performed US-TSI with heating arrays transmitting at 45 V and meanwhile measured the temperature increase. At each location, the temperature measurements were averaged over three repeated measurements. To depict a smooth spatial 2-D temperature increase pattern using these measurements from sparse locations, we applied a biharmonic spline interpolation by using the MATLAB (MathWorks Inc., Natick, MA, USA) function “griddata” with the “v4” method option. For US-TSI, the thermal strain pattern was expected to be almost the same regardless of the axis translations because the gelatin phantom was assumed to be homogeneous. We then averaged the thermal strain image using all the repeated measurements from all axis translations to obtain a robust 2-D thermal strain pattern.

The second objective was to demonstrate that our new transducer can identify and quantify different lipid concentrations, that is, tissue media consisting of water and oil with different ratios. To simulate homogeneous tissue of this type, we fabricated a series of phantoms by combining castor oil, distilled water, gelatin derived from porcine skin, a water-soluble surfactant, an oil-soluble surfactant, guar gum, and cellulose (scatters). We performed underwater US-TSI experiments on each phantom, with 0%, 10%, 30%, and 40% oil concentrations, respectively. The thermocouple was inserted into the phantom and the relative position of the tip was adjusted to locate at the heating focus (at z=25mm and y=0mm) by manipulating the translational stage. US-TSI and temperature measurements were acquired under different heating array transmit voltages, 5, 10, 15, 20, 25, 30, 35, 40, and 45 V. Measurements were repeated 3 times, followed by averaging the processed thermal strain image and temperature increase at focus in each case.

### In vivo pig experiments

**Figure 4.**
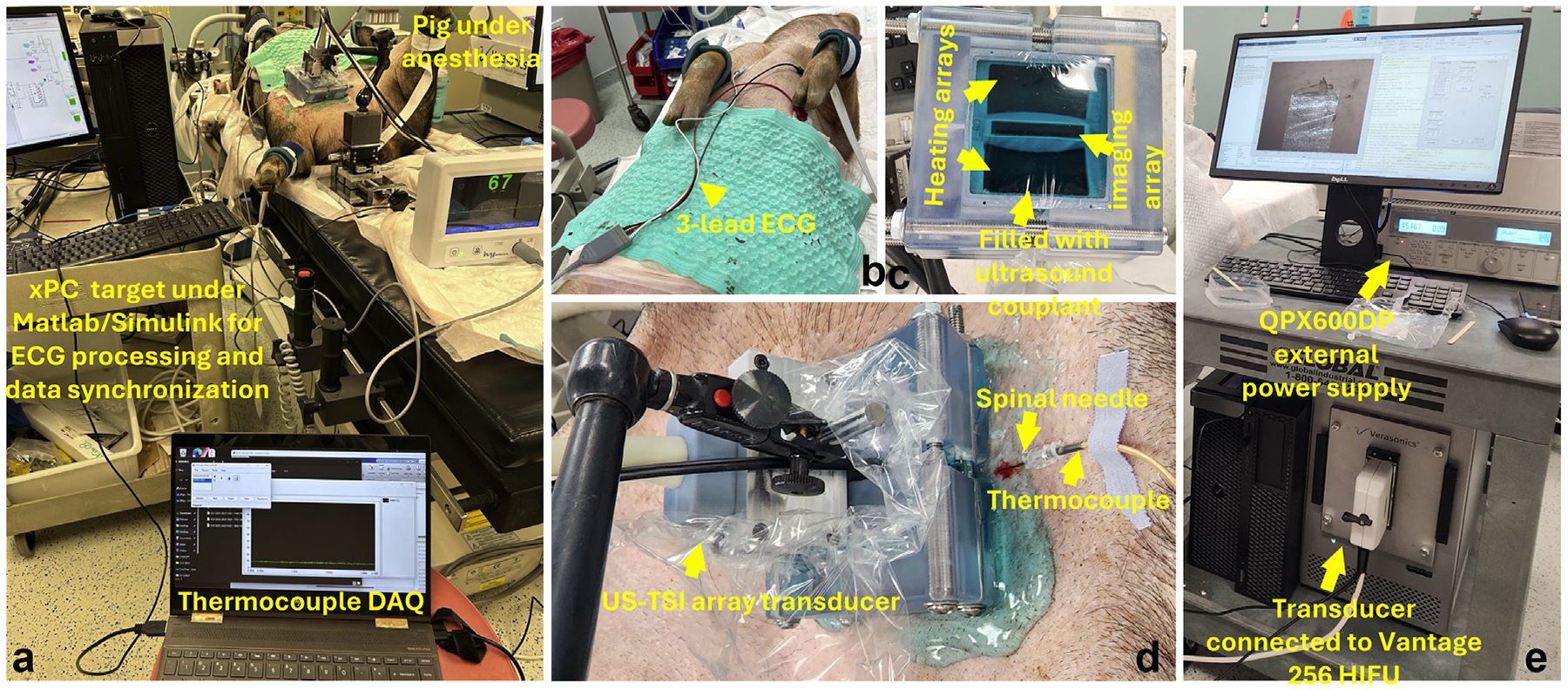
The experiment setup for performing ultrasound thermal strain imaging (US-TSI) *in vivo* on a pig under anesthesia. (a) The overall system for data acquisition, synchronization and triggering US-TSI when the pig is under anesthesia. (b) The three-lead electrocardiogram. (c) Degassed ultrasound couplant applied to the US-TSI array transducer surface for *in vivo* applications. (d) The US-TSI array transducer is fixed by the transducer holder and the thermocouple is inserted through a spinal needle. (e) The US-TSI array transducer is connected to the ultrasound system with an external power supply.

Although direct temperature rise measurement at US-TSI heating focus by thermocouple *in vivo* is almost not practical in human studies owing to invasiveness, by assuming similar tissue properties in the porcine muscle, we performed *in vivo* US-TSI experiments with thermocouple measurements on a Wisconsin Miniature Swine wild-type pig to verify the feasibility of power delivery using our transducer and meanwhile demonstrate the safety. The experiment was approved by the Institutional Animal Care and Use Committee at the University of Pittsburgh. The pig was under anesthesia. Figure 4 illustrates the experimental setup. We first locate a homogeneous area in the vastus medialis muscle near the femoral artery inside the image FOV. Note the term “homogeneous” refers to a spatially uniform tissue characteristic of temperature-dependent SoS change. Under image visual guidance, we inserted a spinal needle (Quincke type, BD) into the thigh to be near the heating focus. The thermocouple was then inserted through the spinal needle until at least 1 cm from its tip was exposed in the tissue and at the heating focus (at z=25mm and y=0mm). A 3-lead ECG device (Ivy Biomedical Systems, Inc., Branford, CT, USA) and a data acquisition system implemented with the QPIDe xPC target (Quanser, Markham, Ontario, Canada) in MATLAB/Simulink (MathWorks Inc.) was used to acquire and process the cardiac signal in real-time. Specifically, we applied real-time finite impulse response bandpass filters to extract the cardiac pulsation and breathing curves and used them as visual feedback. The US-TSI sequence was only triggered during the period when the breathing curve was flat to mitigate the influence from respiratory motions. Once triggered, according to the cardiac pulsation curve, the preheating and post-heating frames were acquired in the phase-matched intervals from two consecutive cardiac cycles and the heating period always started 50 ms before the first post-heating frame. The heating array transmitted at 45 V. The US-TSI was repeated two more times when the thermocouple position was adjusted to (y,z)=(2,25) and (2,25)mm.

## Results

### Phantom experiments to visualize the thermal strain pattern compared with the 2-D temperature map

**Figure 5.**
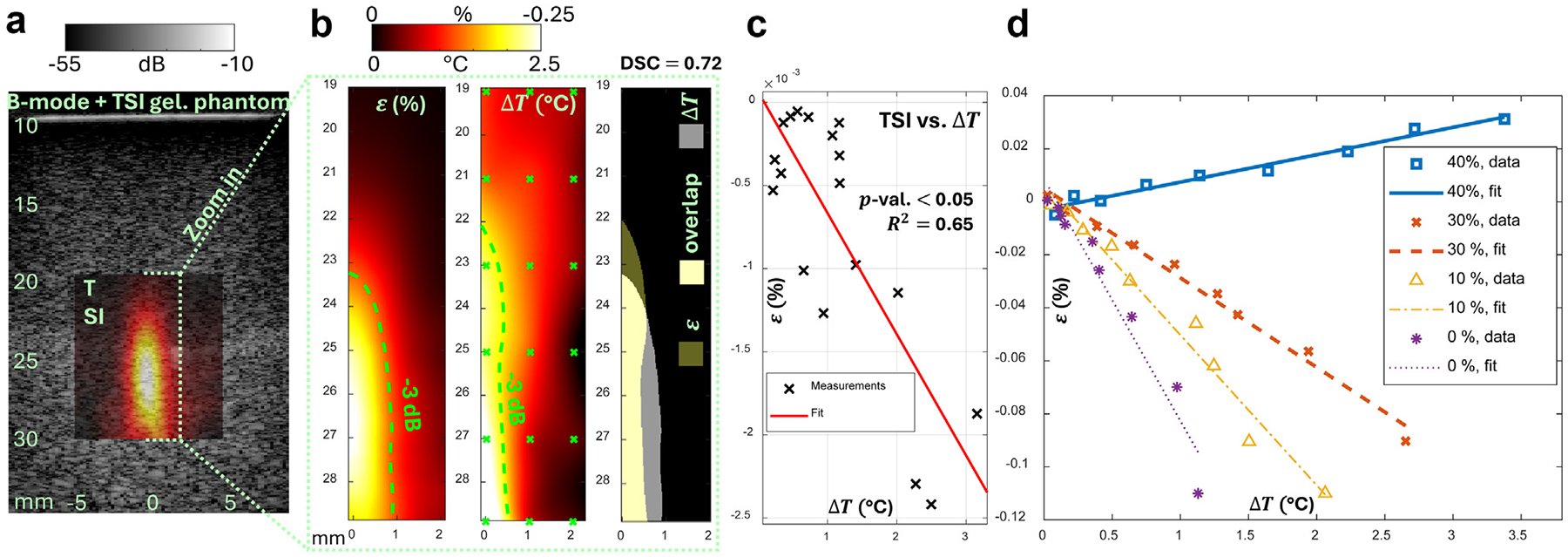
Summary of the results from ultrasound thermal strain imaging (US-TSI) experiments performed on gelatin phantoms underwater. (a) The overlaid ultrasound B-mode image and the co-registered thermal strain image. (b) The comparison between the thermal strain (ε) pattern and the 2-D temperature increase (ΔT) map. The ΔT map was obtained by biharmonic spline interpolation based on the 18 thermocouple measurement points marked by green crosses. The Dice–Sørensen coefficient (DSC) was computed using the −3 dB contour-enclosed regions. (c) Correlation between the thermal strain and the temperature increase indicated by linear regression. (d) US-TSI transducer for quantifying tissue consisting of water and oil with different ratios. The measurements of thermal strain values at heating focus with corresponding temperature increase were produced by different heating array transmit voltages from 5 V to 45V on different phantoms with a different percentage of oil (mass) concentrations.

Figure 5a, 5b, and 5c summarizes the US-TSI and the corresponding distributed spatial temperature increase (ΔT) measurements from underwater experiments on an ultrasound gelatin phantom. The gelatin phantom with 0% oil concentration is considered as all water-bearing tissue and is, therefore, expected to show all negative thermal strains in the target heating region. From the co-registered B-mode and thermal strain (ε) image, we can observe that the peak negative strain reached approximately −0.25%, and the maximum temperature increase was about 2.5°C. To visually compare ε and ΔT, we plotted the −3 dB contour lines and computed the Dice–Sørensen coefficient (DSC) [37] on the regions enclosed by the −3 dB contour lines. We obtained DSC = 0.72 indicating an overlap between two similar patterns. The comparison was further quantitatively assessed by interpolating ε(y,z) to the 18 (y,z) locations where thermocouple measurements, ΔT, are directly available and then computing the correlation between ε(y,z) and ΔT(y,z). The linear regression indicates a good correlation with $R^{2}=0.65$, slope = 0.07, intercept = 0.00, and p<0.05.

### Phantom experiments of US-TSI with different oil concentrations

In addition to evaluating the thermal strain pattern, we also want to demonstrate the capability of the new US-TSI transducer for quantifying tissue consisting of water and oil with different ratios. We performed experiments using a similar setup but on oil-in-gelatin phantoms of different oil concentrations, 0%, 10%, 30%, and 40%. Figure 5d summarizes the results. According to ε=-λΔT, different slopes, -λ, indicate tissues of different oil concentrations. Therefore, we conducted US-TSI with temperature measurements using different heating array transmit voltages and then performed linear regression between ε and ΔT at focus. The result indicates overall good correlations with $R^{2}=0.95,\;0.98,\;0.99,\;0.97$, slope = −0.09, −0.06, −0.03, and 0.01, intercept = 0.01, 0.01, 0.01, and −0.00, respectively, for each oil concentration and p<0.05 for all cases.

### In vivo pig experiments

**Figure 6.**
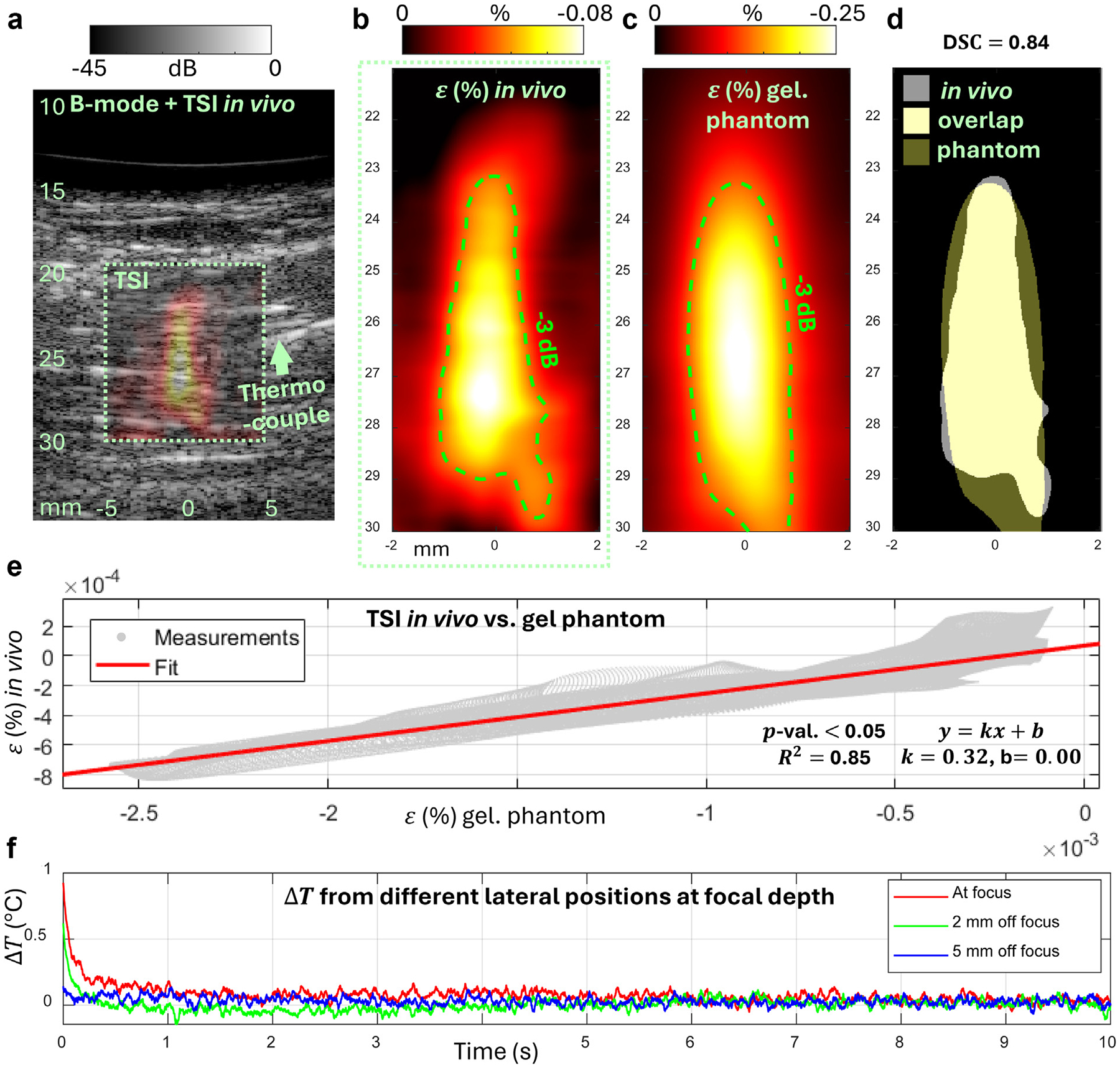
Summary of the results from ultrasound thermal strain imaging (US-TSI) experiments performed *in vivo* and compared with gelatin phantom experiments. (a) The overlaid ultrasound B-mode image and the co-registered thermal strain image. (b) The thermal strain (ε) pattern *in vivo*. (c) The thermal strain (ε) pattern on gelatin phantom. The −3 dB (*green dashed*) contour lines depict the focused heating region. (d) Dice–Sørensen coefficient (DSC) computed using the −3 dB contour-enclosed regions. (e) Linear regression indicating correlation between the thermal strain from *in vivo* pig experiments and that from gelatin phantom experiments. (f) Temperature measured at different lateral locations to the heating focus during *in vivo* pig experiments.

According to the animal model and the postexperiment histology results, the pig was not on a high-fat diet and did not develop lipids. Therefore, we expected the US-TSI performed on the homogenous muscle area to have a similar strain pattern and corresponding temperature measurements compared with the underwater phantom (0% oil concentration) experiments. We can then consider the results from phantom experiments as a benchmark and compare them with the pig experiment results to evaluate the *in vivo* feasibility and performance, including power delivery and safety of the new US-TSI transducer. Figure 6 summarizes the results. The co-registered B-mode and thermal strain (ε) image shows that the overall *in vivo* strain pattern was very similar to that from phantom experiments, which is further confirmed by the −3 dB contour lines in Figure 6b and 6c. The *in vivo* negative peak reached approximately −0.08%, which was reduced to approximately one-third from phantom cases. A more quantitative evaluation computed the DSC and obtained a high score of 0.84 using the −3 dB contourenclosed region (Fig. 6d), as well as a linear regression to calibrate ε(y,z) between phantom and *in vivo* experiments using derived strain values from all the spatial pixel locations, (y,z). Figure 6e presents a very good correlation result of $R^{2}=0.85$, slope = 0.32, intercept = 0.00, and p<0.05.

*In vivo* temperature measurements are also important to evaluate power control and safety. We, therefore, invasively used the thermocouple in the pig experiment to acquire post-heating temperature curves at the focal depth but with different relative lateral locations with respect to the heating focus. Figure 6f presents the temperature curves in a 10 second post-heating time interval. The temperature curves reflected the cooling process from the initial maximum increased temperature, which we used to perform the US-TSI, to the baseline (ΔT=0). The maximum increased temperature was about 0.9°C at focus and about 0.6°C at the location 2 mm laterally away from the focus. Almost no temperature change was measured at 5 mm laterally to the focus.

## Discussion

Visual comparison, DSC score, and linear regression analysis demonstrated consistency among the thermal strain patterns and temperature measurements from gelatin phantom and *in vivo* pig experiments. Particularly, the matched ε and ΔT produced at least 6 mm (axially) by 2 mm (laterally) focused region with peak values in a reasonable numerical range (−0.25%, 2.5°C in phantom and −0.08%, 0.9°C *in vivo*). These demonstrate that the transducer prototype, beamforming, and sequence design can deliver enough acoustic power to effectively heat a wide enough region of interest to produce detectable thermal strain signals by applied image processing methodologies. In addition, by considering temperature increase under a certain heating exposure period as a reasonable metric that reflects how much energy was absorbed by the tissue, those measured peaks of ΔT (around the focus) owing to a precisely program-controlled 50-ms heating interval immediately followed by a 1 to 2 second cooling process back to the baseline temperature can evidence the guarantee of safety [38].

In addition to the effectiveness of power delivery and safety, these experimental results also showed strong linear relationship, ε=-λΔT. that matches the derived US-TSI theory, where the slope –λ is determined by tissue type in terms of oil concentration. As a result, different tissue types can be identified by linear regression using multiple (ε,ΔT) measurements subject to different heating transmit voltages, as in Figure 5d. However, a simpler approach, for example, to only use a single transmit voltage, may be preferred when applied in practice *in vivo*. In this way, the current US-TSI theory can only classify tissue regions above a certain oil concentration as positive strains in contrast to the rest as negative strains. Therefore, sensitivity and specificity calibrations are needed in future studies. Continuing efforts to establish a more comprehensive model of ε=-λΔT to include attenuation or absorption effect and the modeling of the λ term, etc., are also necessary to better calibrate the tissue contrast for *in vivo* applications.

Although the ε pattern *in vivo* highly resembled that from gelatin phantom experiments, the overall magnitude was greatly reduced, as was quantified by the linear regression in Figure 6e with a slope of 0.32. The dominant factors contributing to this could be severe energy loss owing to reflections and attenuations considering the pig skin, subcutaneous layers and muscles. Consistently, the ΔT measured at focus was also reduced from 2.5°C on phantom to 0.9°C *in vivo*. The ratio, computed as 0.9/2.5 = 0.36, is considered close to the slope, 0.32. In addition, the reduction in ε magnitude was spatially consistent everywhere inside the heating area. These suggest that an increased transmit voltage (but under safety pressure limit according to mechanical index < 1.9) is desired for *in vivo* applications to produce strong enough thermal strain signals to be detected by current image processing methodologies.

There were some discrepancies when comparing the ε pattern to the ΔT map on the gelatin phantom, as in Figure 5b. At shallower depths, especially at locations laterally farther away from the center, ΔT showed stronger signals than ε. We suspect these signals were artifacts from thermocouple measurements likely including electromagnetic interference, remaining local mechanical perturbation owing to the excitation by the focused heating beam, and so on, and these effects were mitigated at a greater depth where the thermocouple was farther away from the transducer. In addition, the depth where the peak occurred in the ΔT map (approximately 27 mm) was approximately 1 mm larger than ε (approximately 26 mm). The dimension of the −3 dB-indicated focal region also approximately had a lateral difference of 0.3 mm and an axial difference of 1 mm. Because ΔT and ε were averaged results from repeated trials, with each individual showing close numerical values, the measurements were considered robust. Therefore, we suspect the dimensional discrepancies in the pattern were due to the 2-D interpolation using a coarse mesh grid, that is, only 18 temperature measurement locations. To keep consistent experimental conditions by avoiding extensively long total experiment duration, limited measurement locations restricted the step size to as large as Δy=1mm and Δz=2mm, thus affecting the accuracy of reconstructing the 2-D temperature map using interpolation. Although these discrepancies did not affect the overall comparison, a better measurement strategy for a small temperature increase in a short duration is required for future study.

Artifacts in thermocouple measurements during *in vivo* experiments, could also be created by perturbations owing to tissue physiological motions. This might contribute to the small drift in the temperature curves in Figure 6f, among which, the green curve measured at 2 mm laterally off focus showed the most noticeable and resulted in a possible overestimated ΔT, since according to the −3 dB contour line from benchtop phantom experiments, the heating effect beyond 1 mm laterally should be less than one-half of that right at focus.

The major technical gap between the current preclinical large animal study and human clinical applications is that we have not yet demonstrated *in vivo* lipid detection in AP without ECG gating, which is considered cumbersome to setup appropriately together with US-TSI in point-of-care clinics. Our current ongoing study is trying to address this using an AP pig model and requires additional signal processing to facilitate the mechanical motion disturbances. This problem was not significant in this work; we used ECG gating for real-time tracing of the pulsation and breathing motion and then selected the static nonaffected frames during the post processing. For clinical translation, after facilitating the ECG-free *in vivo* AP lipid detection problem, we need also to make the US-TSI transducer more compact considering better prototype housing and assembly design and meanwhile maintain the power transmission capability. The integrated transducer can then be connected to a clinical ultrasound scanner with an additional external power supply unit that would be able to compactly attach to the ultrasound imaging system.

## Conclusion

The US-TSI experiments performed using our new transducer showed highly correlated patterns among thermal strain patterns on ultrasound gelatin phantoms and *in vivo* pig muscular tissue and the corresponding temperature measurements. The capability of performing US-TSI using the array transducer to identify the contrast between oil and water contents was also demonstrated. The *in vivo* feasibility and safety were further demonstrated by the pig experiments. These results indicate the effectiveness and safety of our new array transducer that has the potential to help the translation of US-TSI to assess lipid in AP to diagnose rupture-prone cases.

## Supplementary Material

1

Supplementary material associated with this article can be found in the online version at doi:10.1016/j.ultrasmedbio.2025.05.016.

## Data Availability

Data are available upon reasonable request.
