# Supplementary material for: Experimental Evaluation of an Array Transducer for Ultrasound Thermal Strain Imaging: Phantom and In Vivo Studies
Source: Ultrasound Med Biol. Author manuscript; Available in PMC 2026 May 28. (PMC13216864; doi:10.1016/j.ultrasmedbio.2025.05.016)
Supplement: 1 [file NIHMS2175708-supplement-1.pdf]

Supplementary Materials

S1. Principle of US-TSI

The time, τ, when the echo signal is received from an ultrasound axial location, z, considering the round trip, can be expressed[1] as $\tau(z) = 2\int_0^z \frac{1}{c(T(z),z)} dz$, where c is the local SoS that also depends on $T(z)$, the local temperature. The corresponding spatial location, $Z(z)$, on the reconstructed ultrasound image from z is then $Z(z) = c_0 \int_0^z \frac{1}{c(T(z),z)} dz$, where c_0 is the constant SoS used during beamforming. The change of object location and image location is then related by $\frac{dZ}{dz} = \frac{c_0}{c(T(z),z)}$. According to these, the current US-TSI theory model the shift on image, $\Delta Z(z)$, of the echo received from location, z, due to a local temperature increase, $\Delta T(z)$, from the baseline temperature $T_0(z)$, as $\Delta Z(z) = c_0 \int_0^z \left(\frac{1+\beta(z)\Delta T(z)}{(1+\lambda(z)\Delta T(z))c(T_0,z)} - \frac{1}{c(T_0(z),z)} \right) dz$. $\beta(z)$ and $\lambda(z)$, determined by local tissue characteristics, linearly approximate the effect of thermal expansion and temperature-dependent SoS change[1], respectively, around $T_0(z)$. Since ΔZ behaves like the "displacement" on the ultrasound image, by using the expression of $\frac{dZ}{dz}$, the thermal "strain" due to temperature increase around $T_0(z)$ in image coordinate can be computed by the spatial gradient, as $\varepsilon(Z) = \left.\frac{d(\Delta Z)}{dZ}\right|_{T_0(z)} = \left.\frac{d(\Delta Z)}{dz}\frac{dz}{dZ}\right|_{T_0(z)} = c_0 \frac{(\beta(z)-\lambda(z))\Delta T(z)}{(1+\lambda(z)\Delta T(z))c(T_0,z)} \frac{c(T_0,z)}{c_0} = \frac{(\beta(z)-\lambda(z))\Delta T(z)}{1+\lambda(z)\Delta T(z)}$. Given small temperature increase (< 10 °C), it can be assumed that thermal expansion is negligible and also assumed[1] that $|\lambda(z)\Delta T(z)| \ll 1$. Then $\varepsilon(Z)$ can be further approximated as $\varepsilon(Z) = -\lambda(z)\Delta T(z)$, which is shown to be determined by the local characteristics and temperature change in tissue. Particularly, provided a well-controlled temperature increase, ΔT, the fact that $\lambda < 0$ in fat and $\lambda > 0$ in water, respectively, makes it feasible to identify lipid contents in contrast to water-bearing tissue by a positive or negative ε, respectively, from US-TSI.

Reference

1 Simon, C., VanBaren, P. & Ebbini, E. S. Two-dimensional temperature estimation using diagnostic ultrasound. *IEEE Trans. Ultrason. Ferroelectr. Freq. Control* **45**, 1088-1099 (1998). https://doi.org/https://doi.org/10.1109/58.710592
